# Does position of the wrist during cast immobilisation in patients with distal radius fractures affect outcome?

**DOI:** 10.1007/s00068-021-01751-8

**Published:** 2021-08-04

**Authors:** Eva Anna Klazina van Delft, Tamara Geertruda van Gelder, Jefrey Vermeulen, Niels Willem Luitzen Schep, Frank Willen Bloemers

**Affiliations:** 1 Department of Trauma Surgery, Amsterdam UMC, VU University Medical Center, Amsterdam Movement Sciences, Boelelaan 1117, Postbox 7057, 1007 MB Amsterdam, The Netherlands; 2 Department of Emergency Medicine, Máxima Medisch Centrum, De Run, 5504 DB Veldhoven, The Netherlands; 3 Department of Trauma Surgery, Maasstad Ziekenhuis, Maasstadweg 21, 2079 DZ Rotterdam, The Netherlands

**Keywords:** Conservative treatment, Distal radial/radius fractures, Cast position

## Abstract

**Purpose:**

The position of the wrist during cast immobilisation following closed reduction of distal radius fractures is disputed. A systematic review was initiated to assess if there was any relation between wrist position in the cast and outcome in adult patients with non-operatively treated distal radius fractures.

**Methods:**

A comprehensive search was performed in the bibliographic databases Medline, Embase and Wiley/Cochrane Library from inception up to 27 November 2020. Eligible studies were: randomised controlled trials, prospective and retrospective comparative cohort studies, analysing different positions of the wrist in cast-immobilisation following closed reduction. Primary outcome of the study was functional outcome measured by range of motion. Secondary outcomes were functional outcomes measured by grip strength, patient-reported outcome, radiological outcome and complications such as secondary dislocation and pain.

**Results:**

The initial search yielded 2733 studies. Five trials, with 519 patients, were included in this systematic review. Range of motion and radiological outcome was significantly better in patients who were immobilised in dorsiflexion compared to palmar flexion or neutral position, although no clinical important difference was found. There were no significant differences in patient-reported outcome, pain, grip strength or complications. Due to heterogeneity of the included studies, data were unsuitable for a meta-analysis.

**Conclusion:**

This systematic review showed statistically significant better results in favour of cast immobilisation in dorsiflexion, although this small difference does not seem to be relevant in patients daily activities.

**Systematic review registration number:**

Systematic review registration number: PROSPERO 2018 CRD42018085546.

**Supplementary Information:**

The online version contains supplementary material available at 10.1007/s00068-021-01751-8.

## Introduction

Nearly 20% of all fractures are distal radius fractures (DRFs) [1]. Most of these fractures are treated non-operatively with closed reduction and cast immobilisation.

Previous studies have shown no superiority of above-elbow casts compared to lower forearm casts [2–4]. However, the best position of the wrist in lower forearm casts, remains controversial. Some authors believe that dorsiflexion (DF) of the wrist prevents fracture displacement since DF balances the forces of the radial extensors and flexors best [5–7]. Some conclude that pronation is more effective in retaining the reduction, others state that supination prevents fracture displacement [8–11].

The Cochrane review “Conservative interventions for treating distal radial fractures in adults” [12], analysed six articles comparing different wrist positions during cast immobilisation and showed no significant difference in clinical, functional or anatomical outcome [7, 11, 13]. Since then, three new trials were published that compared DF to palmar flexion (PF) or neutral position (NP) [6, 14, 15]. No new trials on supination were published after 1990.

The aim of this systematic review was to assess if there is any relation between wrist position in the cast and outcome in adult patients with non-operatively treated DRFs.

## Materials and methods

A review protocol (PROSPERO 2018 CRD42018085546) was developed based on the “Preferred Reporting Items for Systematic Reviews and Meta-Analyses” statement [16].

A comprehensive search was performed in the bibliographic databases Medline, Embase and Wiley/Cochrane Library from inception up to 27 November 2020, in collaboration with a medical librarian. The following terms were used (including synonyms and closely related words) as index terms or free-text words: “Conservative Treatment”, “Non-operative”, “Casts”, “Radius Fractures”, “Adults”. The search was performed without date or language restriction. After deduplication all titles were screened and appropriate abstracts reviewed. Also, a manual reference check of the identified systematic reviews and meta-analyses was executed. The full search strategies for all databases can be found in the supplementary Information. After deduplication, all titles and abstracts were screened independently by two reviewers (ED, TG).

### Eligible criteria

Randomised controlled trials, prospective and retrospective comparative cohort studies comparing different positions of the wrist during cast immobilisation of displaced and reduced DRFs in adults were included in this study. Studies had to report on patient-reported, functional or radiological outcome. Studies were included if they compared wrist immobilisation in DF versus PF or NP and had to define the position of the wrist during cast-immobilisation, which had to be at least 15° of DF or PF. If studies contained other fractures than DRFs, or if the studies concerned paediatric fractures or open fractures, they were excluded. All prospective and randomized studies comparing supination, pronation and ulnar deviation (UD) have been published before 1990 and were discussed in the Cochrane review published in 2003, in which it was concluded that these positions had no influence on outcome [12]. Therefore these studies were excluded from this systematic review and only studies comparing DF to PF or NP were included in the present study. Data extracted from the studies included patient demographics, fracture classifications, duration of wrist immobilisation and the number and type of complications. Restrictions on language of publication were not imposed. In case of disagreements, independent judgement of a third author (FB) was initiated.

### Outcome measures

Primary outcome of the study was functional outcome measured by range of motion. Secondary outcomes were functional outcomes measured by grip strength, patient-reported outcome, radiological outcome and complications such as pain and secondary dislocation.

Functional outcome included range of motion or loss of range of motion, measured in degrees. Grip strength was defined by pressure measured with a dynamometer and expressed in kilograms, mmHg or as a percentage value of the uninjured side.

Patient-reported outcome measures had to be presented in validated questionnaires. Appropriate questionnaires were the Disability of Arm, Shoulder and Hand score (DASH), the Patient Reporting Wrist Evaluation score (PRWE) and the SF-12 Healthy Survey. The PRWE and DASH questionnaires both result in a score between 0 and 100, bases on solely subjective outcomes; 0 is the best possible outcome and 100 is the worst [17, 18]. The SF-12 is a short version of the SF-36 Health Survey, reporting on physical and mental health on a 12-point scale [19, 20]. The Modified Demerit Score and the Sarmiento Score were also qualified. These are scoring systems combining both objective and subjective patient factors as disability, pain, stiffness, range of motion, grip strength and complications as arthritis and nerve dysfunction. The patient-reported outcome is expressed as poor, fair, good or excellent [21].

Radiological outcome was assessed by measuring radial height or radial shortening, dorsal tilt, volar tilt and ulnar variance. Additionally, the Lidström-score was used to determine displacement in a severe, moderate, small or insignificant rating based on the severity of radial shortening, dorsal tilt, and loss of radial inclination [22].

Statistical significance will be analysed, as well as the minimal clinically important difference (MCID). This is the smallest change in outcome that could be identified by an individual patient during daily activities and exercise [23–25].

### Risk of bias assessment and quality scoring

The Cochrane Risk of Bias Tool was used to assess the Risk of Bias (RoB). This tool reflects on seven types of bias. A judgement is made whether the article is at ‘Low’ risk, ‘High’ risk, or ‘Unclear’ risk of bias. If a study fulfilled four or more criteria it was considered as low RoB. Analysis of performance bias was disregarded in this systematic review, because blinding for position of the cast was inapplicable. To give authors the opportunity to provide feedback, attempt was made to contact the authors with the RoB assessment. Although, no attempt was made to contact authors for publications before the year 2000.

### Statistical methods

Review Manager software, version 5.3, Cochrane Collaboration, London, UK, was used to carry out the statistical analysis. Population and study protocol were reviewed to determine clinical homogeneity. Statistical homogeneity was determined by use of the *I*_2_ test, visual inspection of forest plots and by use of the *Q*-test. An *I*_2_ test with values less than 40% and a *Q*-test with *p* < 0.05 was considered to present no significant heterogeneity. Whenever possible, data will be pooled to perform a quantitative analysis.

## Results

**Fig. 1:**
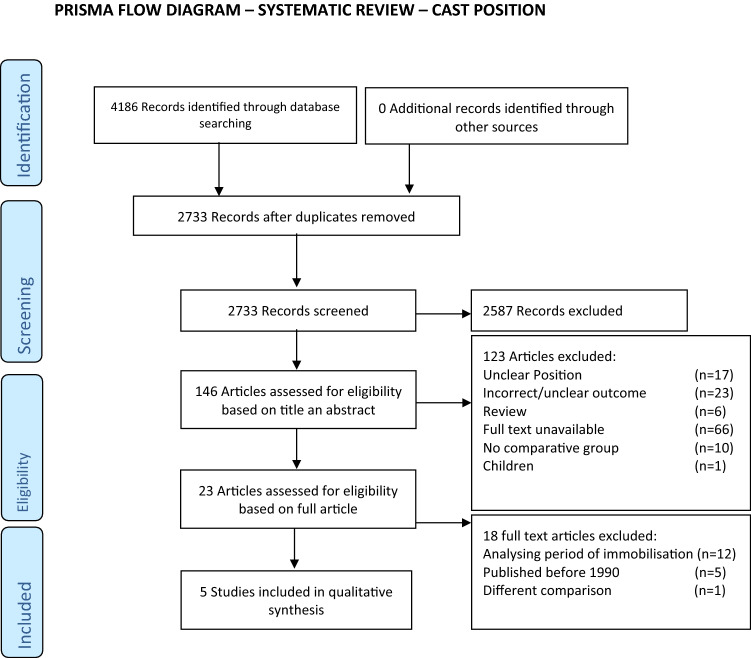
Prisma flow diagram

The primary selection of articles resulted in 146 eligible articles. 141 Articles were excluded, as shown in Fig. 1 [16]. Five articles comparing DF to PF or NP were analysed in this systematic review, including 519 patients (Table 1). There were 348 females, mean age: 56 years. 209 patients were treated in DF, 195 in PF and 115 in NP.

**Table 1:** Articles eligible for systematic review

	Author	Year	Article	*n*	Mean FU	Study design			Risk of bias	Level of evidence	Intra-/extra-articular
						Group I	Group II	Group III			
1	Grle [6]	2017	PC	100	2m	DF	PF	–	Unclear	II	Both
2	Gupta [7]	1991	PC	204	5m–2y	DF	PF	NP	Unclear	II	Both
3	Blatter [8]	1994	RCT	50	2–7y	DF	NP	–	Unclear	II	Both
4	Grafstein [14]	2010	RCT	101	6m	DF	PF	NP	Low	II	Not specified
5	Rajan [15]	2008	PC	64	6m	DF	PF	–	High	II	Extra
	Total			519							

*RCT* randomised controlled trial, *PC* prospective cohort, *FU* follow-up, *y* years, *m* months, *DF* dorsiflexion, *NP* neutral position, *PF* palmar flexion

### Primary outcome

Functional outcome measured by range of motion was analysed in three studies (Table 2) [6, 8, 15]. All three studies showed statistically significant differences in range of motion in favour of patients immobilised in DF.

**Table 2:** Range of motion

Author	Outcome	Outcome			*p* value
		DF	PF	NP	
Range of motion					
Grle [6]	PF	63.60°	64.90°	–	NS
	DF	60.70°	53.90°	–	NS
	RD	17.80°	14.80°	–	< 0.05
	UD	29.00°	24.40°	–	< 0.001
Blatter [8]	Loss of pronation	0.6°	–	3.8°	NS
	Loss of supination	1.2°	–	3.24°	NS
	Loss of PF	2.0°	–	9.2°	< 0.01
	Loss of DF	3.0°	–	5.2°	NS
	Loss of RD	0.6°	–	1.4°	NS
	Loss of UD	0.4°	–	4.2°	< 0.01
Rajan [15]	> 50° pronation	97.1%	90%	–	NS
	> 50° supination	88.2%	70%	–	0.05
	> 30° PF	100%	63.3%	–	< 0.05
	> 45° DF	100%	43.3%	–	< 0.05
	> 15° RD	82.4%	53.3%	–	< 0.05
	> 15° UD	97.5%	70%	–	< 0.05

*PF* palmar flexion, *DF* dorsiflexion, *NP* neutral position, *RD* radial deviation, *UD* ulnar deviation, *NS* not significant

### Secondary outcome

Four studies compared grip strength (Table 3) [6, 8, 14, 15]. Grip strength was significant better in one study favouring DF [15].

**Table 3:** Grips strength

Author	Outcome	Outcome			*p* value
		DF	PF	NP	
Grip strength					
Grle [6]	Strength	49.5 mmHg	43.4 mmHg	–	NS
Blatter [8]	Loss of grip strength in mmHg*	3.8 mmHg	–	6.2 mmHg	NS
Grafstein [14]	Loss of grip strength in Kg*	7–10 kg			NS
Rajan [15]	> 2/3 recovery of grip recovery*	76.5%	23.3%	–	< 0.05

*PF* palmar flexion, *DF* dorsiflexion, *NP* neutral position, *NS* not significant

*Compared to uninjured side

The patient-reported outcome was measured in only two studies (Table 4) [6, 14]. The study of Grle reported significant better SF-12 scores in favor of immobilisation in DF [6].

**Table 4:** Patient-reported outcome

Author	PROM	Outcome			*p* value
		DF	PF	NP	
Grle [6]	PRWE	27.13 SD ± 22.5	25.87 SD ± 20.1	–	NS
	SF12	43.10 SD ± 8.4	39.26 SD ± 7.0	–	< 0.01
Grafstein [14]	DASH 8w	Mean 34.6			NS
	DASH 6m	Mean 20.3			NS

*PROM* patient-reported outcome measure, *PF* palmar flexion, *DF* dorsiflexion, *NP* neutral position, *w* weeks, *m* months, *NS* not significant

Three studies determined outcome by use of a combination of patient-reported and functional outcome: the Modified Demerit Score and the Sarmiento Score, of which two reported significant better outcome in favor of immobilisation in DF (Table 5) [7, 8, 15].

**Table 5:** Combined patient-reported/functional outcome

Author	PROM	Outcome			*p* value
		DF (%)	PF (%)	NP (%)	
Gupta [7]	G/E-outcome*	86	67	62	UK
	P/F-outcome*	14	33	38	UK
Blatter [8]	G/E-outcome*	96	–	80	< 0.05
	P/F-outcome*	4	–	20	< 0.05
Rajan [15]	G/E-outcome*	91.2	66.7	–	< 0.05

*PROM* patient-reported outcome measure, *PF* palmar flexion, *DF* dorsiflexion, *NP* neutral position

*Derivative of the Modified Demerit Score or Sarmiento score: *G/E-outcome* good or excellent outcome, *P/F-outcome* poor or fair outcome, *UK* unknown

All five studies reported on radiological outcome [6–8, 14, 15]. The studies of Grafstein and Gupta did not report significant difference in radiological outcome [7, 14]. Three studies reported an overall statistically significantly better radiological outcome in patients treated in DF (Table 6) [6, 8, 15].

**Table 6:** Radiological outcome

Author	Outcome	Outcome			*p* value
		DF	PF	NP	
Grle [6]	RH	10.2 mm	9.1 mm	–	< 0.01
	DT	2.7°	3.2°	–	NS
	RI	20.0°	17.3°	–	< 0.01
Blatter [8]	RS	0.58 mm	–	1.64 mm	< 0.01
	DT	4.16°	–	11.7°	< 0.05
	RI	3°	–	4.52°	< 0.05
Rajan [15]	% patients with RI 13–33°	73.5%	46.7%	–	< 0.01
	% patients with VT 1–21°	67.6%	30%	–	< 0.01
	% patients with UV − 2–0 mm	64.7%	40%	–	< 0.05

*PF* palmar flexion, *DF* dorsiflexion, *NP* neutral position, *RH* radial height, *RS* radial shortening, *DT* dorsal tilt, *RI* difference in radial inclination, *VT* volar tilt, *mm* millimetres, *UV* ulnar variance, *NS* not significant

Complications were reported in three studies [6, 14, 15]. Only 1 study described an overall rate of secondary displacement of 22% (*n* = 22), of which 17 were treated operatively. The difference in secondary displacement between DF (*n* = 5), PF (*n* = 8) and NP (*n* = 9) was not significant (*p* = 0.17) [14].

Pain was described in two studies [6, 14]. One study reported significant decrease in pain in the patients treated in DF compared with immobilisation in PF [6]. Persistent pain at 6-month follow-up was reported in one patient in each group [14].

One study described carpal tunnel syndrome in two patients, one in the DF group, one in the NP group [14]. Another study described an increased rate of Complex Regional Pain Syndrome (Morbus Sudeck) in the DF group comparing to the PF group, however the number of patients suffering from complications, neither the significance was reported [6]. The third study reported three patients with complaints of stiffness in the PF group, yet none of the patients showed complaints at the final follow-up [15].

### Risk of bias within studies

**Fig. 2:**
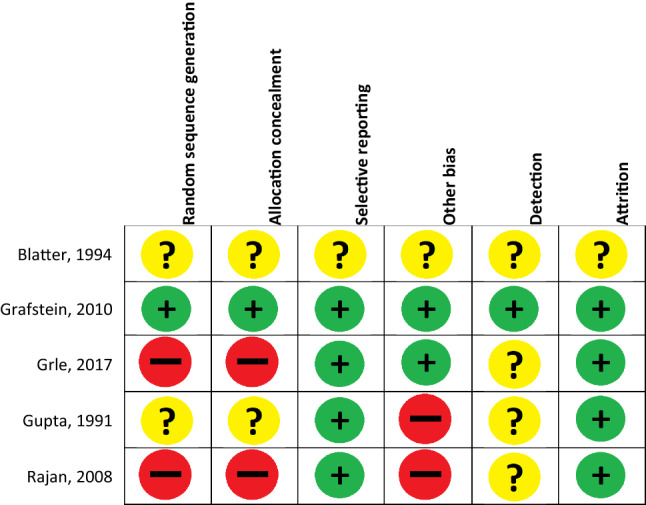
Risk-of-bias summary: reviewers judgments regarding each risk-of-bias item for the included studies according to the Cochrane Risk of Bias Tool

Using the Cochrane Risk of Bias Tool, one study was qualified as low-risk study, one as high-risk study and in three of the studies the risk of bias was unclear (Table 1; Fig. 2).

## Discussion

A systematic review was initiated to assess the optimal position of the wrist during cast immobilisation in patients with non-operatively treated DRFs.

Our review showed that patients treated with cast immobilisation in DF had a range of motion or radiological outcome that was statistically significantly better according to patients who were immobilised in PF or NP. Nevertheless, the clinical relevance of this statistical difference is limited. Grip strength, patient-reported outcome, or rate of complications were not statistically different between the different positions of cast-immobilisation.

Differences found in our study were small, and despite significant results were found, the results were too small to reach minimal clinically important difference (MCID). This is the smallest change in outcome that could be identified by an individual patient during daily activities and exercise [26–28]. Unlike patient-reported outcome measures as the DASH and PRWE, and grip strength, the MCID could not be determined for radiological parameters or range of motion. Nevertheless, differences in radiological outcome and range of motion found in this study were that small that clinical importance is assumed to be negligible.

The results of this review were limited by the risk of bias in studies and the strength of available evidence. Studies used non-validated measurements to report their outcome. Grip strength should be reported as a percentage of the unaffected side [29]. Also, the Modified Demerit Score and Sarmiento score are, in contrast to the DASH and PRWE, not validated instruments. To report pain, by example, not all studies used the validated VAS-score [30, 31]. Most of the included studies presented their results without describing mean values or standard deviations, resulting in data that could not be pooled to perform a quantitative analysis by meta-analysis.

Also, studies did not use a standardized follow-up period. The follow-up period in the study of Grle was only 2 months. Plausibly, grip strength and range of motion will improve further after this period of time [6].

Besides the use of different, non-validated, measuring tools, there was no homogeneity in fracture types too. The studies included in this review analysed both intra and extra articular DRF. No subgroup analysis bases on fracture type were performed in any of the studies. One can presume that patients with a more comminuted fracture will have a worse anatomical and functional outcome compared to a simple fracture.

To draw clear conclusions on the best position of immobilisation in DRFs, future studies should use homogeneous patient data and validated outcome measures and report standard deviations to enable quantitative analysis.

This systematic review showed statistically significant better results in favour of cast immobilisation in dorsiflexion, although these differences are too small to be noticed for patients during their daily activities.

## Supplementary Information

Below is the link to the electronic supplementary material.

Supplementary file1 (DOCX 17 kb)
