# Supplementary material for: Does position of the wrist during cast immobilisation in patients with distal radius fractures affect outcome?
Source: Eur J Trauma Emerg Surg. 2021 Aug 4;48(3):1751–7. doi: 10.1007/s00068-021-01751-8 (PMC9192448; doi:10.1007/s00068-021-01751-8)
Supplement: Supplementary file 1 — Supplementary file1 (DOCX 17 kb) [file 68_2021_1751_MOESM1_ESM.docx]

**Supplementary Information (27 November 2020)
PubMed Session Results**

| Search | Query | Items found |
| --- | --- | --- |
| [#11](https://www.ncbi.nlm.nih.gov/pubmed) | #9 NOT #10 | [1,](https://www.ncbi.nlm.nih.gov/pubmed/?cmd=HistorySearch&querykey=11)554 |
| [#10](https://www.ncbi.nlm.nih.gov/pubmed) | head[ti] OR neck[ti] | [124,](https://www.ncbi.nlm.nih.gov/pubmed/?cmd=HistorySearch&querykey=10)883 |
| [#9](https://www.ncbi.nlm.nih.gov/pubmed) | #8 NOT "Case Reports"[Publication Type] | [1,](https://www.ncbi.nlm.nih.gov/pubmed/?cmd=HistorySearch&querykey=9)641 |
| [#8](https://www.ncbi.nlm.nih.gov/pubmed) | #7 NOT ("Animals"[Mesh] NOT "Humans"[Mesh]) | [1,](https://www.ncbi.nlm.nih.gov/pubmed/?cmd=HistorySearch&querykey=8)879 |
| [#7](https://www.ncbi.nlm.nih.gov/pubmed) | #6 NOT (("Adolescent"[Mesh] OR adolescen*[tiab] OR "Child"[Mesh] OR "Infant"[Mesh] OR child*[tiab] OR schoolchild*[tiab] OR infant*[tiab] OR girl*[tiab] OR boy*[tiab] OR teen[tiab] OR teens[tiab] OR teenager*[tiab] OR youth*[tiab] OR pediatr*[tiab] OR paediatr*[tiab] OR puber*[tiab]) NOT ("Adult"[Mesh] OR adult*[tiab] OR man[tiab] OR men[tiab] OR woman[tiab] OR women[tiab])) | [1,](https://www.ncbi.nlm.nih.gov/pubmed/?cmd=HistorySearch&querykey=7)932 |
| [#6](https://www.ncbi.nlm.nih.gov/pubmed) | #4 OR #5 | [2,](https://www.ncbi.nlm.nih.gov/pubmed/?cmd=HistorySearch&querykey=6)528 |
| [#5](https://www.ncbi.nlm.nih.gov/pubmed) | #1 AND #3 | 960 |
| [#4](https://www.ncbi.nlm.nih.gov/pubmed) | #1 AND #2 | 2,115 |
| [#3](https://www.ncbi.nlm.nih.gov/pubmed) | wrist*[tiab] AND fracture*[tiab] | 6,140 |
| [#2](https://www.ncbi.nlm.nih.gov/pubmed) | "Radius Fractures"[Mesh] OR ("Radius"[Mesh] OR radius[tiab] AND fracture*[tiab]) OR radial fracture*[tiab] OR colles fracture*[tiab] OR barton fracture*[tiab] OR barton’s fracture*[tiab] OR smith fracture*[tiab] OR smith’s fracture*[tiab] OR colles’s fracture*[tiab] | [14,](https://www.ncbi.nlm.nih.gov/pubmed/?cmd=HistorySearch&querykey=2)299 |
| [#1](https://www.ncbi.nlm.nih.gov/pubmed) | "Conservative Treatment"[Mesh] OR conservative[tiab] OR non-operative[tiab] OR non-surgical[tiab] OR nonoperative[tiab] OR nonsurgical[tiab] OR "Casts, Surgical"[Mesh] OR "Splints"[Mesh] OR cast[tiab] OR casts[tiab] OR casting[tiab] OR plaster*[tiab] OR splint[tiab] OR splints[tiab] | 205,448 |

**Embase.com Session Results**

| Search | Query | Items found |
| --- | --- | --- |
| [#11](https://www.ncbi.nlm.nih.gov/pubmed) | #9 NOT #10 | [1,](https://www.ncbi.nlm.nih.gov/pubmed/?cmd=HistorySearch&querykey=11)742 |
| [#10](https://www.ncbi.nlm.nih.gov/pubmed) | head:ti OR neck:ti | [154,](https://www.ncbi.nlm.nih.gov/pubmed/?cmd=HistorySearch&querykey=10)409 |
| [#9](https://www.ncbi.nlm.nih.gov/pubmed) | #8 NOT 'case report'/de | [1,](https://www.ncbi.nlm.nih.gov/pubmed/?cmd=HistorySearch&querykey=9)782 |
| [#8](https://www.ncbi.nlm.nih.gov/pubmed) | #7 NOT ([animals]/lim NOT [humans]/lim) | 2,089 |
| [#7](https://www.ncbi.nlm.nih.gov/pubmed) | #6 NOT (('adolescent'/exp OR 'child'/exp OR adolescent*:ti,ab OR child*:ti,ab OR schoolchild*:ti,ab OR infant*:ti,ab OR girl*:ti,ab OR boy*:ti,ab OR teen:ti,ab OR teens:ti,ab OR teenager*:ti,ab OR youth*:ti,ab OR pediatr*:ti,ab OR paediatr*:ti,ab OR puber*:ti,ab) NOT ('adult'/exp OR 'aged'/exp OR 'middle aged'/exp OR adult*:ti,ab OR man:ti,ab OR men:ti,ab OR woman:ti,ab OR women:ti,ab)) | 2,126 |
| [#6](https://www.ncbi.nlm.nih.gov/pubmed) | #4 OR #5 | [2,](https://www.ncbi.nlm.nih.gov/pubmed/?cmd=HistorySearch&querykey=6)610 |
| [#5](https://www.ncbi.nlm.nih.gov/pubmed) | #1 AND #3 | 1,211 |
| [#4](https://www.ncbi.nlm.nih.gov/pubmed) | #1 AND #2 | [2,](https://www.ncbi.nlm.nih.gov/pubmed/?cmd=HistorySearch&querykey=4)028 |
| [#3](https://www.ncbi.nlm.nih.gov/pubmed) | wrist*:ab,ti,kw AND fracture*:ab,ti,kw | 7,644 |
| [#2](https://www.ncbi.nlm.nih.gov/pubmed) | 'distal radius fracture'/exp OR (('distal radius'/exp OR radius:ab,ti,kw) AND fracture*:ab,ti,kw) OR 'radial fracture*':ab,ti,kw OR 'colles fracture*':ab,ti,kw OR 'barton fracture*':ab,ti,kw OR 'barton s fracture*':ab,ti,kw OR 'smith fracture*':ab,ti,kw OR 'smith s fracture*':ab,ti,kw OR 'colles s fracture*':ab,ti,kw | [12,](https://www.ncbi.nlm.nih.gov/pubmed/?cmd=HistorySearch&querykey=2)956 |
| [#1](https://www.ncbi.nlm.nih.gov/pubmed) | 'conservative treatment'/de OR conservative:ab,ti,kw OR 'non-operative':ab,ti,kw OR 'non-surgical':ab,ti,kw OR nonoperative:ab,ti,kw OR nonsurgical:ab,ti,kw OR 'plaster cast'/exp OR 'splint'/exp OR cast:ab,ti,kw OR casts:ab,ti,kw OR casting:ab,ti,kw OR plaster*:ab,ti,kw OR splint:ab,ti,kw OR splints:ab,ti,kw | 291,059 |

**Cochrane Library Session Results**

| Search | Query | Items found |
| --- | --- | --- |
| [#6](https://www.ncbi.nlm.nih.gov/pubmed) | #4 OR #5 | 581 |
| [#5](https://www.ncbi.nlm.nih.gov/pubmed) | #1 AND #3 | 326 |
| [#4](https://www.ncbi.nlm.nih.gov/pubmed) | #1 AND #2 | 496 |
| [#3](https://www.ncbi.nlm.nih.gov/pubmed) | (wrist* and fracture*):ab,ti,kw | 931 |
| [#2](https://www.ncbi.nlm.nih.gov/pubmed) | ((radius and fracture*) or (radial NEXT fracture*) or (colles* NEXT fracture*) or (barton* NEXT fracture*) or (smith* NEXT fracture)):ab,ti,kw | 1,644 |
| [#1](https://www.ncbi.nlm.nih.gov/pubmed) | (conservative or (non NEXT operative) or (non NEXT surgical) or nonoperative or nonsurgical or cast or casts or casting or plaster* or splint or splints):ab,ti,kw | 17,525 |
